# Women's Future in Medicine: Achievements and Opportunities

**Published:** 1915-09-04

**Authors:** 


### WOMEN'S FUTURE IN MEDICINE.

### Achievements and Opportunities.

To-day few regard the woman doctor as an innovation in our social and professional life, though formerly she was considered, at any rate by the majority, as an unwelcome intruder in a sphere of work in which tradition had allotted her no place. For in recent times public opinion, not only regarding women in medicine, but as to their place in the general work of the world, has undergone a complete change. It is now recognised that in most of the professions women are destined to practise side by side and on equal terms with men. Yet, as time goes, it is only a short space since Addison wrote of the occupations of women that "The sorting of a suit of ribands is reckoned a good morning's work; and if they make an excursion to a mercer's or a toy shop, so great a fatigue makes them unfit for anything else all the day after. Their more serious occupations are sewing and embroidery, and their greatest drudgery the preparation of jellies and sweetmeats." What the essayist wrote applied, of course, to the state of ordinary women, for, as he knew, there were multitudes of those of a more elevated life and conversation, that moved in an exalted sphere of knowledge and virtue, that joined all the beauties of the mind to the ornaments of dress, and inspired a kind of awe and respect into their male contemporaries.

### The Early Days of the Movement.

It is less than fifty years since the pioneers of women in medicine—the late Miss Elizabeth Blackwell and Mrs. Garrett Anderson, who is happily still with us—were entered on the British Medical Register. For many years the movement that had been inaugurated by these two women and Dr. Sophia Jex-Blake was very slow, and, like all endeavours to introduce reforms that are contrary to established custom, it met with no outside encouragement, all the force required to keep the movement alive being provided by the pioneers themselves. And now, after a little more than forty years, women have an assured place in medicine, and the successful nature of their work is being manifested in several very remarkable ways. To say that the war brought women their opportunity would be to create a wrong impression, for their opportunity came long before the war, and they were prepared and did not fail to seize it. Already they were doing valuable work, not only in private practice, but in health departments, in sanitation, in Poor-Law institutions, and in hospitals. The sphere of their work was not confined to this country, for they were already engaged in medical missions, while a considerable body of medical women were attached to hospitals in India; and some idea of their success in that Empire can be obtained from the statement in the recently issued Blue Book on the moral and material progress of India, that many provinces are paying special attention to increasing the supply of women doctors, and that a school of medicine for women in the Punjab is being assisted by a Government grant; moreover, in the medical colleges of Bombay, Madras, Calcutta, Lahore, and Lucknow there are in all considerably over a hundred women students. Already Englishwomen were also practising in Africa, the United States, Canada, the West Indies, the Argentine, British Guinea, Korea, the Malay Peninsula, Palestine, Australia, and New Zealand. But, although women doctors had an established position in the profession long before the war, there can be no doubt that the war has brought with it much vaster opportunities than there had ever been before, and greatly enlarged their field of usefulness. Had there been four times the present number of medical women there would have been scope for every one of them for professional employment, and one can only regret that in the earlier days of the movement public encouragement was so scanty; had it been otherwise we might have had in our hour of need two or three thousand registered women instead of the thousand who are now in practice.

### A Great Achievement.

The call to arms immediately took thousands of medical men from their practices and from the hospitals, and, fortunately for the health of the community, qualified women were in readiness to take the place of a large proportion of those who had gone on active service. But this is by no means the outstanding feature of women's work in the present crisis. That women were prepared to take the places vacated by men is in itself a striking fact, but they have opened up new fields for themselves, and, without waiting for any call other than that of suffering humanity, they have done wonderful work among the wounded soldiers, concerning most of which the world will learn nothing until long after the war is over. Within a few weeks of the commencement of hostilities Dr. Flora Murray and Dr. Garrett Anderson organised a women's hospital corps and took it to France; this was the first of the voluntary medical units to receive patients in the early days of September last year. In due course the unit was removed to Boulogne, where their work was so successful that the War Office invited Dr. Murray and Dr. Anderson to come to London and there organise a large military hospital to be conducted by women only. This was by far the greatest undertaking of the kind with which women had ever been confronted, but the magnitude of the task was not so great as their zeal or their capabilities. A huge block of buildings in Endell Street, Holborn, which was formerly a workhouse, was placed at their disposal, and, although the preparations did not begin until the end of February 1915, the buildings were fully equipped and a great hospital of 520 beds was fully staffed and ready to receive patients by May 12. When it is realised that at the time these two medical women commenced their task the buildings were still furnished as a workhouse, that all this furniture had to be removed, that structural alterations on a large scale had to be made, that the wards and hospital throughout had to be equipped and a full staff engaged, this may be fittingly described as a colossal achievement. The doctor in charge is Dr. Flora Murray, and the medical staff consists of fourteen women doctors, including the visiting staff; there is a woman quartermaster, and there are thirty-six nursing sisters, eighty women orderlies, as well as stewards and storekeepers. We believe this is the only official military hospital conducted by women, and that never before has there been a woman in charge of a military hospital or a woman quartermaster. The staff is paid by the War Office, and there are no voluntary workers. It is difficult to find words to express our admiration of the wonderful work which Dr. Murray and her staff are doing. In connection with the training of medical women the hospital is likely to play an important part, for it offers a unique opportunity for gaining experience of a kind that has not been available to women. Hitherto there have been few facilities for women to deal with major traumatic surgery, but this hospital provides a splendid occasion for such experience. It should be added that most of the beds are for surgical cases, and that consequently this great undertaking not only serves as a wonderful illustration of what women can do, but opens up new opportunities in medical education.

### What Medical Women are doing in France.

Although the hospital in Endell Street is by far the largest of the kind conducted by women, there are, of course, voluntary military institutions which are under the control of medical women. For instance, there was established at Troyes some three months ago a unit of the Scottish Women's Hospitals, and this is worked in connection with the French War Office. This hospital, which has been equipped mainly at the expense of present and past students of Girton and Newnham Colleges, contains two hundred beds. It is situated in the grounds of the Château Chanteloup, about a mile from the town of Troyes, the administrative staff being lodged in the Château itself. The sick and wounded soldiers are accommodated in marquees holding ten to sixteen beds, each of the tents being so made that the sides can be rolled up during the day time. The "orangerie" has been converted into an operating theatre, and *x*-ray apparatus has been installed in the saddlery. The institution is, in fact, equipped in the style of a modern hospital, but it has the advantage over town hospitals in that it affords a means of treatment in the open air.

### Medical Education for Women.

It is thus shown that the future for women in medicine is exceedingly promising. This might have been truly said before the war, but it can now be stated more emphatically. The question as to how a woman can qualify as a doctor is easily answered, by the advice to pass a suitable preliminary examination and enter one of the medical schools available for women. For geographical and other reasons the majority of women would probably enter the London (Royal Free Hospital) School of Medicine for Women. When this school was founded in 1874 by Dr. Sophia Jex-Blake it occupied a small private house in Hunter Street, Brunswick Square, and to begin with there were fourteen students. Larger premises on the original site were built in 1900, and were opened by King Edward and Queen Alexandra (then Prince and Princess of Wales). The school has now outgrown these buildings, for there are to-day well over two hundred students, and a further entry of nearly seventy is expected next term. When the school was founded there was no general hospital which would admit women students to its wards for clinical experience, but the Royal Free Hospital has not only thrown open its wards to women students, but has made qualified women eligible for resident and visiting appointments. Forty years ago there was no qualifying body which would examine women for its degrees. Now the London School of Medicine for Women is a School of the University of London in the Faculty of Medicine, and all other examining bodies, except the Universities of Oxford and Cambridge, admit women to their qualifying examinations. The applications for entrance are so numerous that the council has found it necessary to appeal for funds to provide an extension to the school building. A piece of land which adjoins the present premises has been secured, and the extension will comprise enlargements of the present laboratories, new research rooms, and new lecture rooms. The cost of the extension, with its equipment, is estimated at £30,000, and of this sum £12,000 has been subscribed.

It is hoped that the new premises will be completed by next spring, when there will be accommodation for seventy-five new students. The course of training for the medical degrees of the University of London occupies 5½ years, and for all other qualifications the minimum time of study is five years. In the provinces most of the medical schools are open to women, and degrees are granted to them at all the four Scottish Universities—Edinburgh, Glasgow, St. Andrews, and Aberdeen. In Glasgow there is a school of medicine for women only, which is part of the University, and there is a school of medicine for women at Edinburgh. Apart from positions held by women in military hospitals, many valuable appointments are open to them in time of peace, and at the present time medical women are acting as assistant medical officers, medical inspectors in various Government Departments, resident medical officers in infirmaries, asylums, and hospitals, medical inspectors in secondary schools, lecturers on hygiene and public health, medical officers in quarantine stations, and pathologists in various public and hospital laboratories. We think we have said enough to show that women are destined to play an important part in the future, and that medicine offers them vast opportunities for usefulness and many prizes.

